# Association of a new 99-bp indel of the *CEL* gene promoter region with phenotypic traits in chickens

**DOI:** 10.1038/s41598-020-60168-2

**Published:** 2020-02-21

**Authors:** Xiangnan Wang, Xinlei Wang, Bingjie Chen, Yaping Guo, Hehe Tang, Donghua Li, Danli Liu, Yanbin Wang, Guoxi Li, Xiangtao Kang, Zhuanjian Li, Ruili Han

**Affiliations:** College of Animal Science and Veterinary Medicine, Henan Agricultural University, Zhengzhou, 450046 China

**Keywords:** Gene amplification, DNA replication

## Abstract

Carboxyl ester lipase (CEL) encodes a cholesterol ester hydrolase that is secreted into the duodenum as a component of pancreatic juice. The objective of this study was to characterize the *CEL* gene, investigate the association between the *CEL* promoter variants and chicken phenotypic traits, and explore the *CEL* gene regulatory mechanism. An insertion/deletion (indel) caused by a 99-bp insertion fragment was shown for the first time in the chicken *CEL* promoter, and large differences in allelic frequency were found among commercial breeds, indigenous and feral birds. Association analysis demonstrated that this indel site had significant effects on shank length, shank girth, chest breadth at 8 weeks (*p* < 0.01), evisceration weight, sebum weight, breast muscle weight, and leg weight (*p* < 0.05). Tissue expression profiles showed extremely high levels of the *CEL* gene in pancreatic tissue. Moreover, the expression levels of the genes *APOB*, *MTTP*, *APOV1* and *SREBF1*, which are involved in lipid transport, were significantly reduced by adding a 4% oxidized soybean oil diet treatment at the individual level and transfecting the embryonic primary hepatocytes with a *CEL*-overexpression vector. Interestingly, the results showed that the expression level of the II homozygous genotype was significantly higher than that of the ID and DD genotypes, while individuals with DD genotypes had higher phenotypic values. Therefore, these data suggested that the *CEL* gene might affect body growth by participating in hepatic lipoprotein metabolism and that the 99-bp indel polymorphism could be a potentially useful genetic marker for improving the economically important traits of chickens.

## Introduction

Carboxyl ester lipase (CEL), also known as cholesterol esterase or bile salt-dependent lipase, is attributed to its ability to hydrolyze multiple lipids and is a lipolytic enzyme with broad substrate specificity^1,2^. The enzyme is primarily synthesized and expressed in large quantities in lactating mammary glands and in the exocrine pancreas and is stored in zymogen granules, which are then activated by bile salts in the intestine^3–5^. After consumed feed reaches the gastrointestinal tract, the enzyme is released into the intestine, mainly as a supplement to other lipolytic enzymes. Although it constitutes only 1–5% of the pancreatic enzyme content, it has an important effect on the hydrolysis of lipid nutrients in dietary fat^6–8^. In addition, CEL secreted by the liver and present in plasma not only contributes to the assembly and secretion of chylomicrons but also participates in low-density lipoprotein (LDL) lipid metabolism, the selective uptake of cholesterol esters and reverse cholesterol transport in high-density lipoprotein (HDL)^9–11^.

According to the National Center for Biotechnology Information (NCBI) database, the *CEL* gene contains 11 exonic sequences in human, bovine, murine, chicken and other vertebrate genomes. Interspecific comparisons of genes and proteins have shown that the *CEL* gene retains essentially similar properties, structures and key sequences across species^12^. Previous studies have shown that variable numbers of tandem repeat (VNTR) mutations and copy number variants (CNVs) located in *CEL* exon 11 can lead to MODY-pancreatic exocrine syndrome^3,13,14^, alcoholic cirrhosis^15^, insulin-dependent diabetes mellitus or noninsulin dependent diabetes mellitus^13^, pancreatic cancer^16^, alcoholic pancreatitis and other pancreatic exocrine diseases^17,18^.

The above facets of the role and mutation of *CEL* in mammalian species have been extensively studied; however, although high *CEL* expression in layer chickens has been found only in pancreatic tissues^19^, no research on *CEL* gene mutations in chickens and livestock has yet been reported.

Insertion/deletion (indel) is one of the main sources of molecular-level evolutionary variation, and has been used widely as a major molecular marker in the study of economic traits in livestock and poultry^20,21^. Chickens are an economically important poultry species, prompting great interest in improving the qualitative and quantitative traits of poultry^22^. Previous association studies have determined that a 9–15 bp indel locus in the premelanosome protein (*PMEL*) gene is responsible for a disease-causing mutation in feather color^23^, and dwarfism can be caused by a deletion mutation in the 3’ untranslated region (3’ UTR) of the growth hormone receptor (*GHR*) gene^24^.

In the current study, the main objectives were to analyze the relationship between the novel 99-bp insertion mutation in the *CEL* gene promoter and growth/carcass traits of chickens, and to explore the biological effects of different allelic variants of the *CEL* gene and regulatory mechanisms in hepatic lipoprotein metabolism. These results could improve the understanding of the *CEL* regulatory mechanisms in chickens, suggesting a molecular marker that may facilitate genetic selection and thereby improve poultry performance.

## Results

### Conserved synteny analysis and cloning of the chicken CEL gene

**Figure 1:**
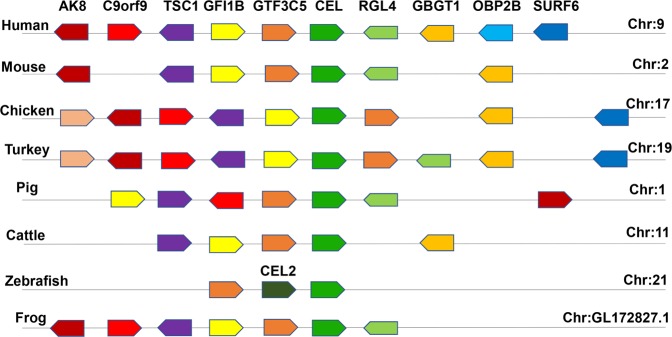
Genetic conservation analysis of the *CEL* gene in different species. Note: Species are listed on the left side of the chromosome, and gene symbols are listed at the top. Boxes of the same color refer to genes that are identical in different species. The orientation of the boxes of different genes represents their direction of transcription.

The *CEL* gene is located on chromosome 17, contains 11 exons and 10 introns and encodes 556 amino acids (NP_001013015.1) in chickens. We performed conserved synteny analysis on different species, demonstrating that *CEL* in chicken and other species is positioned at the same locus of a conserved genomic region containing common genes including *TSC1*, *GFI1B*, *GTF3C5*, *RGL4*, and *GBGT*1 (Fig. 1). The full-length CDS region was obtained using Lushi blue-shell (LS) chicken pancreas cDNA as a template (Supplementary Fig. S1). The results of DNA sequence analysis showed that the number of A bases was 419 (25.07%); G, 426 (25.49%); C, 454 (27.17%); T, 372 (22.26%); A + T, 791 (47.34%); and G + C, 880 (52.66%) and that the molecular weight of the protein was 61.1391785 kDa. Bioinformatics analysis results revealed that there is a Pfam Abhydrolase_3 functional domain at amino acids 121 to 230. There is a signal peptide and a possible transmembrane domain from amino acids 5–27 in the CEL protein sequence with the N-terminus located inside the cell and the C-terminus outside. Moreover, there are three potential N-linked glycosylation (Asn-X-Ser/Thr) sites at amino acid positions 207, 270 and 381 in the extracellular domain (Supplementary Fig. S2). These results illustrate the conservation of *CEL* gene sequences in different species.

### Identification of a novel CEL 99-bp indel polymorphism

**Figure 2:**
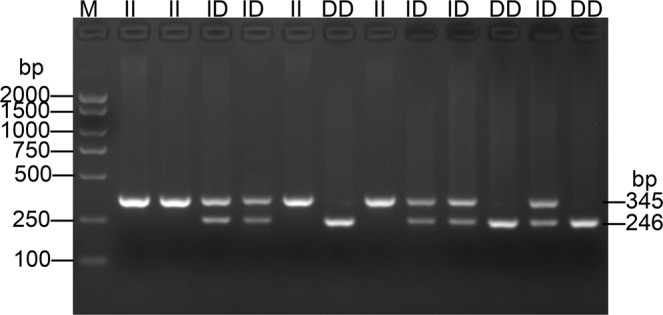
Electrophoresis pattern of the 99-bp indel locus within the chicken *CEL* gene.

**Figure 3:**
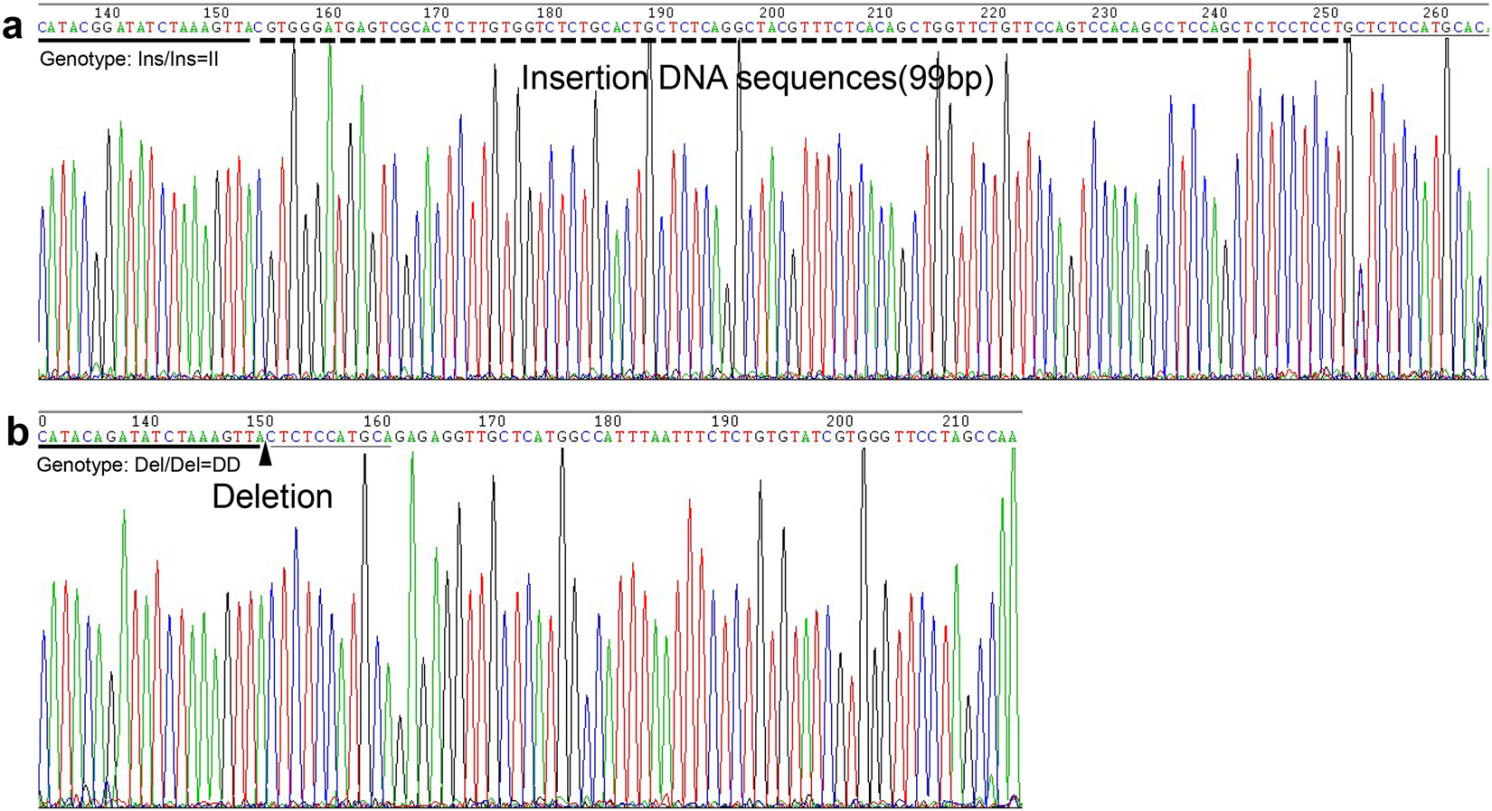
Sequencing comparison of a novel 99-bp indel polymorphism of promoter region within the chicken *CEL* gene. Note that as the triangle symbol shows, the 99-bp indel could be easily noticed on the dotted line. The underlined nucleotides at the beginning of the sequences represent similarities between the “insertion” variant and “deletion” variant.

A new 99-bp indel (NC_006104.4:g.7244459insCGTGGGATGAGTCGCA CTCTTGTGGTCTCTGCACTGCTCTCAGGCTACGTTTCTCACAGCTGGTTCTGTTCCAGTCCACAGCCTCCAGCTCTCCTCCTG7244460) polymorphism of the *CEL* gene was revealed by whole genome resequencing of five Xichuan black-bone (XC) individuals. The indel variants, with the respective alleles being I and D, were genotyped by 2.0% agarose gel electrophoresis in different chicken breeds. As illustrated in Fig. 2 (cropped from Supplementary Fig. S3), three genotypes were observed, including genotype II, genotype ID and genotype DD. Allele I is 246-bp, while the length of allele D is 345 bp (Supplementary Fig. S4). Compared with published chicken genome sequences, the PCR product sequencing result suggests that a 99-bp insertion is located in a region 4310 bp upstream of the *CEL* gene (Fig. 3). This polymorphic sequence has been deposited in the Database of Genomic Variants (DGVa accession number: estd240).

### Population genetic analysis of the indel in 10 chicken populations

Table 1 shows the distribution of genotypic and allelic frequencies of 99-bp indels in feral, local and commercial chicken populations, as well as their genetic diversity, which will help clarify when this insertion mutation occurred during chicken domestication. Large differences in allelic frequency were found across feral birds and indigenous and commercial breeds. For the Chinese indigenous breeds, which are characterized by slow growth and a history of undergone nonsystematic selection, we found that the frequency of the insertion allele was approximately 35.20% to 46.20%. In contract, in highly directionally selected commercial chicken breeds we found low insertion allele frequencies (3.00% to 9.90%). The results of a χ2 test showed that the 99-bp indel was in Hardy-Weinberg equilibrium (HWE) (*p* > 0.05) in the tested chicken breeds. In addition, the difference in the distribution of genotypes among commercial breeds was significant (*p* < 0.01), but nearly no significant difference was observed among the indigenous breeds (Supplementary Table S1). The values of *He* and *Ne* were 0 to 0.497 and 1 to 1.988, respectively. According to the classification of *PIC*, the indigenous chicken breeds exhibit moderate polymorphism (0.250 < *PIC* < 0.500), whereas commercial chickens exhibit low polymorphism (*PIC* < 0.250). Pairwise *Fst* values also indicated a moderate degree of genetic differentiation between commercial and native breeds (0.05 < *Fst* < 0.26). A higher differentiation value (more than 0.25) was found between Changshun blue-shell (CS) chickens and Ross 308, suggesting that the insertion mutation was highly selected in modern meat-type chickens. Among the commercial breeds, there was little differentiation, and *Fst* values among indigenous chickens were less than 0.02, indicating no obvious differentiation (Table 2). These results suggest that this insertional mutation locus may undergo low selection pressure during the evolutionary history of the indigenous breeds and is more strongly selected in recent artificially selected commercial chickens.

**Table 1:** Population genetic analysis of the *CEL* 99-bp indel polymorphism in 10 chicken breeds.

Breeds	N	Genotypic and allelic frequencies						*He*	*Ne*	*PIC*	*p*-value (*HWE*)
		*II*	*ID*	*DD*		*I*	*D*				
F_2_	794	0.306	0.523	0.171		0.567	0.433	0.491	1.964	0.370	0.068
XC	185	0.195	0.465	0.340		0.427	0.573	0.489	1.958	0.370	0.496
LS	190	0.168	0.474	0.358		0.405	0.595	0.482	1.931	0.366	0.811
CS	144	0.174	0.576	0.250		0.462	0.538	0.497	1.988	0.374	0.056
DX	172	0.134	0.436	0.430		0.352	0.648	0.456	1.838	0.352	0.565
HB	217	0	0.198	0.802		0.099	0.901	0.179	1.217	0.163	0.105
Ross 308	166	0	0.060	0.940		0.030	0.970	0.058	1.062	0.057	0.689
AA broiler	192	0	0.198	0.802		0.099	0.901	0.178	1.217	0.162	0.282
KF	24	—	—	—		0.250	0.750	—	—	—	—
TiC	11	—	—	—		0.364	0.636	—	—	—	—

Note: F_2_, XC, LS, CS, DX, HB, AA, KF, and TiC represent the F_2_ resource population, Xichuan black-bone chicken, Lushi blue-eggshell chicken, Changshun blue-eggshell chicken, Dongxiang blue-eggshell chicken, Hy-Line Brown, Arbor Acres, Kauai feral chicken, and Tibetan chicken, respectively. N = Number of samples; *He* = gene heterozygosity; *Ne* = effective allele numbers; *PIC* = polymorphism information content; *p*-value (*HWE*) = *p*-value of Hardy-Weinberg equilibrium.

**Table 2:** Pairwise fixation index (*Fst*) of the *CEL* gene between indigenous and commercial chickens.

POP	KF	TiC	XC	LS	CS	DX	HB	Ross308
TiC	0.0136							
XC	0.0132	0.0009						
LS	0.0101	0.0004	0.0005					
CS	0.0224	0.0026	0.0012	0.0032				
DX	0.0050	0.0000	0.0059	0.0030	0.0125			
HB	0.0202	0.0324	0.1425	0.1272	0.1711	0.0947		
Ross 308	0.0978	0.1343	0.2157	0.1976	0.2612	0.1655	0.0181	
AA broiler	0.0325	0.0586	0.1122	0.0992	0.1460	0.0761	0.0000	0.0210

Note: KF = Kauai feral chicken, TiC = Tibetan chicken, XC = Xichuan black-bone chicken, LS = Lushi blue-eggshell chicken, CS = Changshun blue-eggshell chicken, DX = Dongxiang blue-eggshell chicken, HB = Hy-Line Brown, AA = Arbor Acres. All *Fst* statistical values were tested by GENEPOP 4.5 with Bonferroni correction. The cutoff value was set at *p* < 0.002 at a 5% confidence interval.

### A 99-bp insertion is correlated with CEL expression

**Figure 4:**
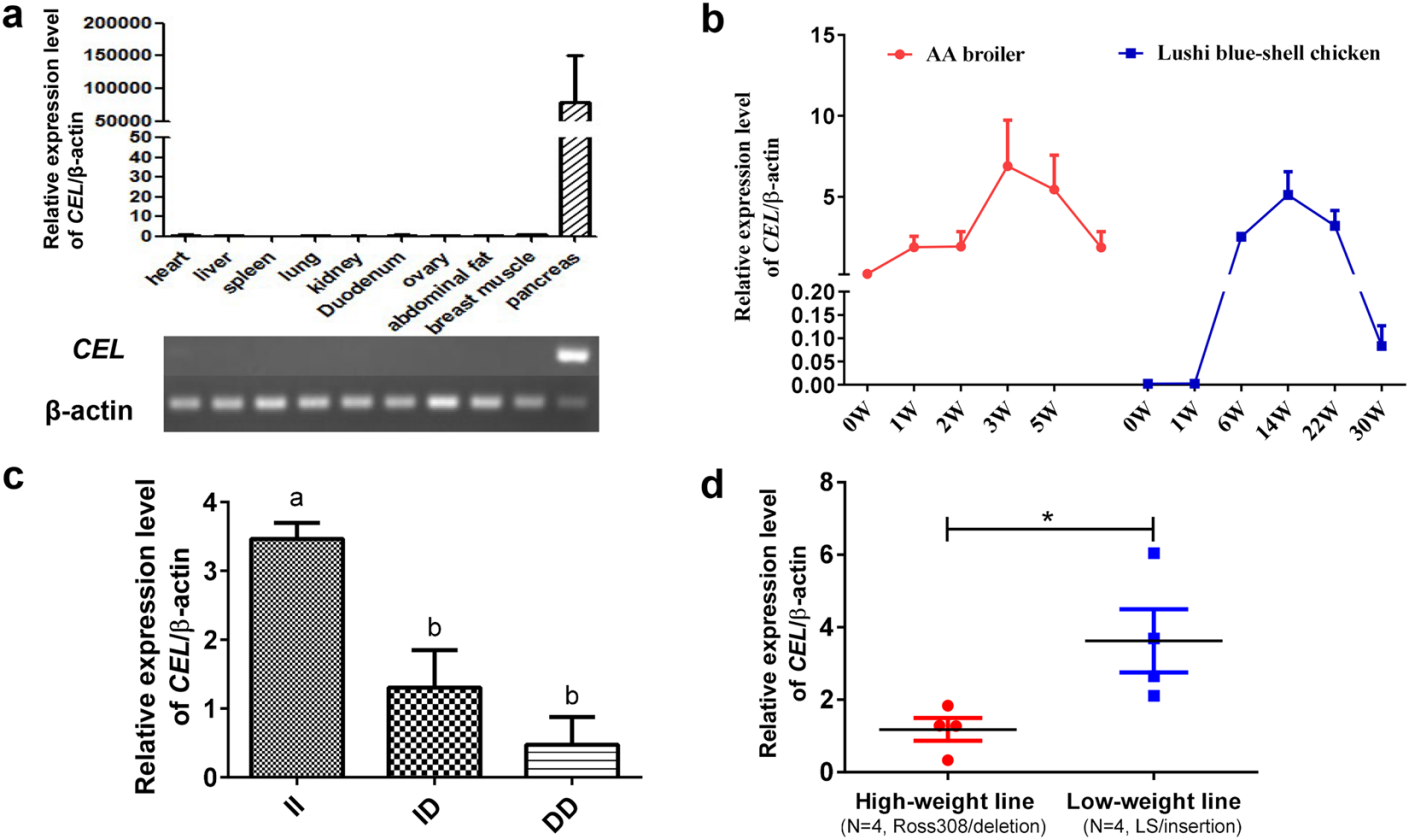
Expression analysis of the *CEL* gene. (**a**) Tissue expression profiles and electrophoresis patterns of the *CEL* gene in 14-week-old Lushi chickens. (**b**) Spatiotemporal expression of the *CEL* gene in pancreatic tissue of AA broiler and Lushi chickens. (**c**) Expression pattern of chicken *CEL* mRNA for various genotypes. Means with different superscripts indicate significant differences: different subscripts indicate *p* < 0.05; and matching subscripts indicate no difference (*p* > 0.05). (**d**) *CEL* expression in the pancreas tissue of high-weight line and low-weight line chickens (N = 4 per line). The high-weight line is from Ross 308 birds with the DD genotype of *CEL*. The low-weight line is the Lushi indigenous breed with a feral II genotype of *CEL*. *p < 0.05 was estimated by Student’s t-test. All data are presented as the means ± SD.

Tissue expression profile analysis showed extremely high levels of the *CEL* gene in pancreas tissues, whereas it displayed hardly any expression in other tissues including the heart, liver, spleen, lung, kidney, duodenum, ovary, abdominal fat, and breast muscle (Fig. 4a, cropped from Supplementary Fig. S5). Additionally, tissue expression profiles of four species also showed extremely high *CEL* gene expression in pancreatic tissue (Supplementary Fig. S6). During chicken growth, we noticed that expression of the *CEL* gene first increased and then decreased in pancreas tissue of Arbor Acres (AA) broilers and LS chickens. The expression of the *CEL* gene was highest at 3 weeks of age and lowest at 0 weeks of age (*p* = 0.002); Due to the long growth cycle of local chickens, we observed the trend of *CEL* gene expression from 0 to 30 weeks of age: the highest expression level was at 14 weeks of age, and the lowest was at 0 weeks (Fig. 4b, *p* = 0.025). Next, we detected *CEL* expression of different genotypes and found that the II genotype showed significantly higher expression than the DD group (*p* = 0.003, Fig. 4c). Interestingly, we also found that *CEL* expression in the II genotype (low-weight line, n = 4) was also much higher than that in the DD genotype (high-weight line, n = 4) (Fig. 4d, *p* = 0.039). Therefore, we suspected that the *CEL* gene may be involved in the rapid growth of chickens and that this insertion mutation contributes to increased expression of *CEL* in chicken pancreas tissues.

### Association analysis of the indel with chicken economic traits

Further association analysis was used to investigate the functional effects of the 99-bp insertion polymorphism in the *CEL* gene on chicken phenotypic traits. In individuals aged 4 to 12 weeks, the 99-bp indel showed significant associations with shank length (SL), shank girth (SG), chest breadth (CB) at 8 weeks, and breastbone length (BBL) at 4 weeks (*p* = 0.005; *p* = 0.007; *p* = 0.007; and *p* = 0.032, respectively). Individuals with ID genotypes had a longer BBL at 4 weeks than the II birds. At 8 weeks old, the SL, SG, and CB of birds with the DD genotype were significantly larger than those of birds with the II genotype (Table 3). Carcass traits directly affect economic value, and we found that the effect of the indel on carcass traits, including semi-evisceration weight (EW), subcutaneous fat weight (SFW), breast muscle weight (BMW) and leg weight (LW), was significant (Table 4) (*p* < 0.05) and that the phenotypic values of DD genotype individuals were the highest. However, the difference between ID and II genotypes were not significant (*p* > 0.05). Furthermore, no association was found between the indel locus and body weight (BW) traits of chickens at ages of 0 and 12 weeks (*p* > 0.05, Supplementary Table S2), and the BW of homozygous DD genotypes was greater than that of homozygous II genotypes. These data implied that this insertion mutation could be associated with growth performance.

**Table 3:** Associations of the *CEL* indel genotypes with the growth traits.

Age	Growth Traits	(Mean ± SE)			*p* value
		II (n = 243)	ID (n = 415)	DD (n = 136)	
0 weeks	SL (cm)	2.58 ± 0.01	2.58 ± 0.01	2.58 ± 0.01	0.809
4 weeks	SL (cm)	5.45 ± 0.06	5.50 ± 0.06	5.54 ± 0.08	0.484
	SG (cm)	2.69 ± 0.03	2.71 ± 0.03	2.70 ± 0.03	0.490
	CD (cm)	4.83 ± 0.04	4.86 ± 0.03	4.79 ± 0.05	0.538
	CB (cm)	4.09 ± 0.04	4.11 ± 0.03	4.07 ± 0.05	0.714
	BBL (cm)	6.15 ± 0.06^b^	6.25 ± 0.05^a^	6.23 ± 0.07^ab^	0.032
	PA (°)	74.22 ± 0.29	74.24 ± 0.25	74.14 ± 0.37	0.963
	BSL (cm)	11.32 ± 0.09	11.41 ± 0.09	11.46 ± 0.1	0.156
	PB (cm)	5.14 ± 0.04	5.14 ± 0.03	5.22 ± 0.04	0.184
8 weeks	SL (cm)	7.82 ± 0.06^b^	7.93 ± 0.05^ab^	8.05 ± 0.07^a^	0.005
	SG (cm)	3.39 ± 0.03^b^	3.44 ± 0.03^a^	3.45 ± 0.03^a^	0.007
	CD (cm)	6.48 ± 0.06	6.55 ± 0.05	6.63 ± 0.08	0.302
	CB (cm)	5.59 ± 0.05^b^	5.73 ± 0.04^a^	5.74 ± 0.06^a^	0.007
	BBL (cm)	8.86 ± 0.07	8.92 ± 0.06	8.98 ± 0.08	0.250
	PA (°)	76.12 ± 0.43	76.63 ± 0.39	76.42 ± 0.51	0.401
	BSL (cm)	16.14 ± 0.09	16.26 ± 0.08	16.34 ± 0.11	0.207
	PB (cm)	6.85 ± 0.06	6.87 ± 0.05	6.93 ± 0.07	0.475
12 weeks	SL (cm)	9.36 ± 0.07	9.41 ± 0.06	9.44 ± 0.07	0.449
	SG (cm)	3.84 ± 0.03	3.85 ± 0.02	3.86 ± 0.03	0.835
	CD (cm)	7.86 ± 0.05	7.88 ± 0.04	7.93 ± 0.07	0.676
	CB (cm)	6.32 ± 0.07	6.35 ± 0.06	6.31 ± 0.08	0.751
	BBL (cm)	10.96 ± 0.08	10.99 ± 0.08	11.02 ± 0.09	0.738
	PA (°)	78.65 ± 0.49	79.15 ± 0.46	79.4 ± 0.54	0.175
	BSL (cm)	19.71 ± 0.09	19.78 ± 0.08	19.92 ± 0.11	0.176
	PB (cm)	8.59 ± 0.06	8.72 ± 0.05	8.73 ± 0.07	0.111

Note: SL = shank length; SG = shank girth; CD = chest depth; CB = chest breadth; BBL = breastbone length; PA = pectoral angle; BSL = body slanting length; PB = pelvis breadth.

Means with different superscripts indicate significant differences: different lowercase letters indicate *p* < 0.05; and the same letters indicate no difference (*p* > 0.05).

**Table 4:** Associations of the *CEL* indel genotypes with the carcass traits in chickens.

Trait	(Mean ± SE)			*p* value
	II (n = 243)	ID (n = 415)	DD (n = 136)	
SEW(g)	1090.31 ± 24.63	1098.36 ± 23.62	1130.20 ± 26.25	0.064
EW(g)	909.55 ± 22.70^b^	918.61 ± 21.90^ab^	946.96 ± 24.02^a^	0.042
SFW(g)	8.16 ± 1.20^ab^	7.37 ± 1.10^b^	10.41 ± 1.37^a^	0.024
BMW(g)	68.38 ± 2.49^b^	70.85 ± 2.41^ab^	72.34 ± 2.63^a^	0.030
LW(g)	148.10 ± 3.89^b^	149.25 ± 3.76^ab^	154.48 ± 4.10^a^	0.030
LMW(g)	98.69 ± 2.91	99.04 ± 2.81	102.09 ± 3.07	0.145
CW(g)	1176.17 ± 25.65	1187.06 ± 24.62	1219.15 ± 27.33	0.057

Note: SEW = semi-evisceration weight; EW = evisceration weight; SFW = subcutaneous fat weight; BMW = breast muscle weight; LW = leg weight; LMW = leg muscle weight; CW = carcass weight.

SE = standard error of the mean; means with different superscripts indicate significant differences: different lowercase letters indicate *p* < 0.05; and the same letters indicate no difference (*p* > 0.05).

### Association analysis of the indel with chicken biochemical variables

Animal blood function is closely related to the body’s metabolism, nutritional status and disease. When animals are in different growth states, the physical and chemical indicators of blood may change accordingly; thus, it is necessary to investigate the impact of the indel mutation on chicken biochemical variables in blood. Association analysis showed that the indel polymorphism was significantly associated with alanine transaminase (ALT), gamma-glutamyl transpeptidase (GGT) and albumin (ALB) (*p* < 0.05). The levels of total cholesterol (TC), triglyceride (TG), HDL and LDL of II genotype birds were higher than those with the DD genotype but no association was found between the indel locus and those traits (Table 5). Collectively, we suggested that this insertion mutation may lead to blood metabolic disorders that affect the growth of chickens.

**Table 5:** Associations of the *CEL* indel genotypes with the biochemical variables in blood investigated in chickens.

Serum variables	(Mean ± SE)			*p* value
	II (n = 243)	ID (n = 415)	DD (n = 136)	
ALT	2.06 ± 0.14^a^	1.80 ± 0.10^ab^	1.32 ± 0.18^b^	0.004
GGT	15.79 ± 0.48^ab^	15.94 ± 0.40^a^	14.44 ± 0.58^b^	0.032
ALB	17.02 ± 0.20^a^	16.55 ± 0.17^b^	16.52 ± 0.24^ab^	0.033
TC	3.20 ± 0.05	3.18 ± 0.04	3.06 ± 0.06	0.101
TG	0.43 ± 0.01	0.41 ± 0.01	0.41 ± 0.01	0.145
HDL	2.001 ± 0.037	1.986 ± 0.032	1.962 ± 0.043	0.671
LDL	1.052 ± 0.031	1.038 ± 0.026	0.971 ± 0.038	0.152

Note: ALT: alanine transaminase, GGT: gamma-glutamyl transpeptidase, ALB: albumin, TC: total cholesterol, TG: triglyceride, HDL: high density lipoprotein, LDL: low-density lipoprotein.

SE = standard error of the mean; means with different superscripts indicate significant differences: different lowercase letters indicate *p* < 0.05; and the same letters indicate no difference (*p* > 0.05).

### Effects of CEL on cholesterol transport *in vivo* and *in vitro*

**Figure 5:**
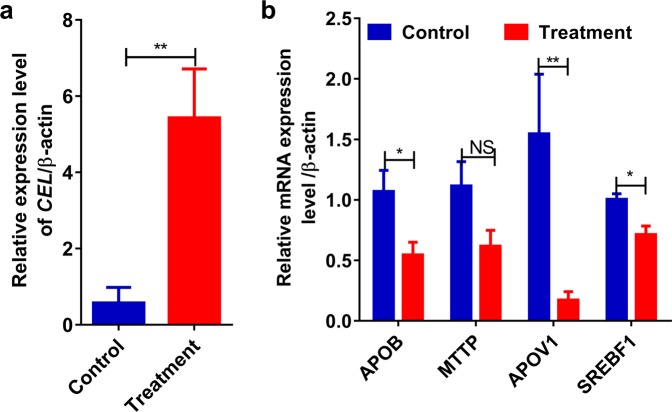
CEL reduces the lipid transport of hepatic lipoprotein metabolism in chicken liver tissue. (**a**) *CEL* expression in the pancreas tissue of the high-fat-diet-fed treatment. (**b**) mRNA levels of *APOB*, *MTTP*, *APOV1* and *SREBF1* in liver tissue were analyzed by qRT-PCR (N = 6). The *ACTB* gene was used as the control. (*p < 0.05 and **p < 0.01).

**Figure 6:**
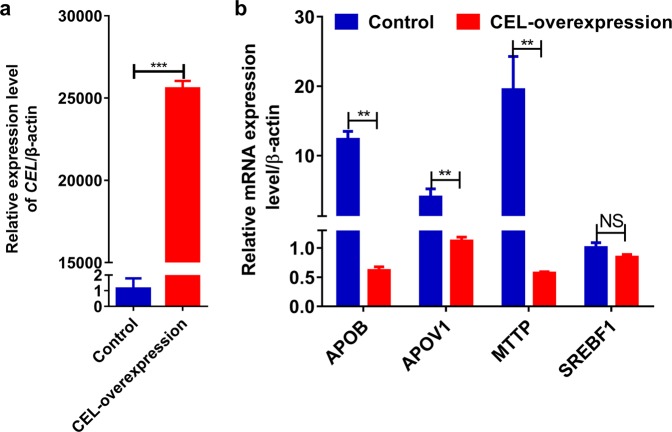
Effect of overexpression of the *CEL* gene on mRNA expression in chicken primary hepatocytes. (**a**) Overexpression of *CEL*. (**b**) mRNA levels of *APOB*, *MTTP*, *APOV1* and *SREBF1* were analyzed by qRT-PCR. The *ACTB* gene was used as the control. (*p < 0.05 and **p < 0.01).

Compared with the control diet, the addition of 4% oxidized soybean oil to the diet showed that the treatment significantly increased the expression of the *CEL* gene (Fig. 5a). Interestingly, the expression levels of the genes *APOB*, *MTTP*, *APOV1* and *SREBF1*, which are involved in lipid transport were significantly reduced (Fig. 5b). Furthermore, *in vitro* treatment results showed that the expression level of *CEL* significantly increased in chicken primary hepatocytes after transfection with pcDNA3.1-CEL-EGFP for 12 h compared with the control condition (Fig. 6a). Additionally, the expression levels of *APOB*, *MTTP*, *APOV1* and *SREBF1* significantly increased in the *CEL*-overexpressing group (Fig. 6b). This result suggests that the *CEL* gene may play a role in hepatic lipoprotein metabolism and regulation of growth by participating in lipid transport.

## Discussion

As important economic traits, growth and body weight performance have become highly important factors in molecular breeding. Because of their simple and convenient characteristics, indel molecular markers have been used widely to improve the economic traits of livestock and poultry. In this study, an indel caused by a 99 bp insertion fragment was shown for the first time in the promoter region of the *CEL* gene. Indel mutations in the promoter region have been found in livestock, suggesting their key role in animal growth and carcass traits^25,26^.

Allelic frequency is used to show the diversity of genes in a population with potential genetic drift, and the novel introduction of mutations^27^. In this study, the genotype frequencies of the *CEL* gene were consistent with the *HWE* (*p* > 0.05) and exhibited low to moderate polymorphism. However, the II genotype is rarely seen and the D allele is the highly predominant allele in commercial breeds, which may be due to the high degree of selection for the *CEL* gene. Artificial selection has produced large phenotypic changes during the domestication of different breeds. Our results indicate that the chicken *CEL* gene has abundant indel polymorphisms in different chicken breeds, and further correlation with growth traits can be analyzed.

Correlation analysis statistical results showed that there were significant effects of the indel on SL, SG, and CW at 8 weeks and on BBL at 4 weeks and that the DD genotype has a higher phenotypic value. Notably, the body shape of chickens is related to bone and muscle development. The direct manifestation of body shape and bone is SL, which is generally used to measure and express bone development. In chickens, the speed of growth is highly correlated with carcass traits and would have a greater impact on economic value^28^. The data associated with the carcass traits indicated that compared with that of the II and ID genotypes, the phenotypic values of the DD genotype were higher in BMW, LW, LMW, CW, SFW, EW, and SEW. Although this mutation had no significant effect on body weight, we still observed that the DD genotype had greater phenotypic values than the II genotype group. This analysis illustrated that the DD genotype is the dominant genotype and that the indel polymorphism can be used as a potentially useful DNA marker.

At present, the protein coding region remains the main focus of human disease research. However, the contribution to variation in the disease does occur in the noncoding region of the genome^29,30^. For example, promoter-specific and enhancer-specific mutations have a clear causal relationship with many Mendelian diseases^31^. Low-density lipoprotein receptor (LDLR) promoter mutations have been shown to cause familial hypercholesterolemia (FH)^32^. In addition, mutations in the core promoter region of the telomerase reverse transcriptase (TERT) gene result in uncontrolled cell proliferation and malignant transformation of hepatocellular carcinoma^33^. In fact, the regulatory mechanism of gene promoter region variation may regulate gene expression by altering transcription factor binding site or promoter activity changes^34^. Studies have shown that the *CEL* gene is highly expressed in the exocrine pancreas with the help of pancreatic transcription factor 1 in humans^5^, mainly due to the presence of the classical TATA and CCAAT elements associated with tissue-specific expression of the gene in the 5’-flanking sequence of the *CEL* gene^35,36^. The tissue expression profile of this study showed that CEL is also highly expressed in the pancreas, and we speculate that this is related to the regulatory elements present in the flanking sequences. In addition, CEL is also present in the liver and blood vessel walls of mammals^37^, and the levels of *CEL* mRNA expression in other tissues including the liver were quite low in the current study. The digestion of fat requires the joint action of bile acids, lipases and colipases; however, the physiological functions of fat digestion in chicks have not yet matured and continue to develop for a few weeks after emergence. The results of this test also show that expression of the *CEL* gene has a low-high-low change trend in AA broiler chickens and local chickens in the pancreatic gland tissues, which was related to the growth rhythm of chickens. Taken together, this evidence suggests that CEL may affect body growth by regulating the body’s lipid metabolism-related pathways.

Previous research indicated that *CEL* is involved in cholesterol esterification, the hydrolysis of dietary fat, and the uptake of fat-soluble vitamins in the duodenum^8^. However, the role of CEL in intestinal cholesterol absorption remains controversial. Cholesterol absorption was shown to be reduced by 80% when pancreas- and bile- diverted rats were supplied with bile and pancreatic juice from which CEL had been removed compared with rats given bile and pancreatic juice containing normal amounts of the enzyme^38^. These results were supported by other studies showing that CEL-specific inhibitors inhibited cholesterol absorption in rats and dogs and reduced serum cholesterol levels in cholesterol-fed rats^39^. In contract, Watt and Simmonds showed that absorption of intestinal cholesterol does not require pancreatic juice, as CEL may remain on the intestinal microvilli after pancreas metastasis^40^. Since cholesterol in the diet and cholesterol in the bile are mainly unesterified and CEL promotes only the absorption of esterified cholesterol by cells^41,42^, the importance of CEL in intestinal cholesterol absorption is not obvious and lack of CEL also does not affect the rate of cholesterol transport from the intestinal lumen to the lymph^10^. Our correlation analysis of biochemical variables in blood showed that TC birds with II and DD genotypes did not show statistically significant differences. In addition to its presence in the digestive tract, Camulli *et al*. first determined that the cytosolic cholesterol esterase present in the liver is CEL, which is identical to the pancreatic enzyme^42–44^, and that CEL is present in specific endosomal compartments of hepatocytes^45^. Winkler *et al*.^46^ showed that hepatocytes can directly secrete hepatogenic CEL. Therefore, the CEL found in the endosomal compartment of the liver may be a recaptured enzyme secreted by hepatocytes, which may play a role in the hepatic secretory capture pathway in which lipoproteins are metabolized^47^. Moreover, the addition of CEL to the culture medium promotes selective uptake of cholesterol esters in HDL by hepatocytes, suggesting a role for CEL in hepatic lipoprotein metabolism^48^. Our results also confirmed that overexpression of CEL in liver primary cells significantly reduced the expression levels of the *APOB*, *MTTP*, *APOV1* and *SREBF1* genes. These results together confirm that CEL may affect the growth traits of chickens by participating in the regulation of hepatic lipoprotein metabolism, which may help understand the mechanism of *CEL* gene regulation in chickens.

In summary, through the identification of a new 99-bp indel of the *CEL* gene promoter region, and analysis of the association of this site with economic traits of chickens, we found significant associations of the indel with chicken growth and development traits. Population genetic analysis revealed that the D alleles are significantly different in the chicken domestication process of different populations, tending to fix in commercial broilers. Further gene expression analysis showed that the gene was highly expressed in pancreatic tissue and participated in the regulation of hepatic lipoprotein metabolism. Collectively, these results will help us understand the CEL regulatory mechanisms of chickens and suggest a molecular marker that may help improve genetic selection, thereby improving poultry performance.

## Materials and Methods

### Ethics statement

All animal experiments were performed according to the Regulations of the Chinese National Research Council (1994) and were approved by the Henan Agricultural University Institutional Animal Care and Use Committee (Permit Number: 11-0085). Chickens were given ad libitum access to feed and water, and all efforts were made to minimize suffering during the experiment^49^.

### Animals and sample preparation

Blood samples were obtained from 2060 individuals including an F_2_ resource population (F_2_, n = 794), four local Chinese chicken breeds (Xichuan black-bone chicken (n = 185, XC), LS chicken (n = 190), CS chicken (n = 144) and Dongxiang blue-shell chicken (n = 172, DX)) and three commercial breeds (Hy-Line Brown hens (n = 217, HB), Ross 308 (n = 166), AA broilers (n = 192)). The F_2_ resource population was constructed via reciprocal crosses between Gushi (GS) chickens and Anka broilers the resulting growth and carcass trait data for 804 F_2_ individuals were recorded using measurement methods detailed in a previous study^50^. We used the growth, carcass and blood metabolic traits of 794 chickens for association analysis. Genomic DNA was isolated from blood samples by the phenol-chloroform method using a commercial kit (Tiangen, Beijing, China), and all DNA samples were diluted to 50 ng/μl and stored at −20 °C for subsequent experiments. In addition, Kauai feral chickens (KF) and Tibetan chickens (TiC) were genotyped using genome resequencing datasets from the NCBI Sequence ReadArchive (SRA) database.

LS chicken is a Chinese indigenous chicken breed well-suited for research involving functional genes in many important biological processes^51,52^, and this is why it was chosen as one of the models to study the regulation of *CEL* gene expression. Twenty 10-week-old LS chickens were randomly divided into two groups and raised to 14 weeks. Among these, ten were fed a complete diet as a control group, and the other 10, the treatment group were additionally given 4 grams of soybean oil per 100 grams of the complete diet. Ten tissue samples (heart, liver, spleen, lung, kidney, duodenum, ovary, abdominal fat, breast muscle, and pancreas) were then collected from all twenty 14-week-old LS chickens within 10 min after slaughter, immediately snap-frozen in liquid nitrogen and stored at −80 °C pending examination of the tissues for the *CEL* mRNA. Furthermore, pancreatic tissues of 14-week-old LS chickens from three individuals of each of the II, ID and DD genotypes were used to detect the *CEL* mRNA expression of different genotypes, and 8 more birds from the Ross 308 and LS breeds (n = 4 per population) were also sacrificed for the same purpose. To further investigate whether the *CEL* gene is involved in lipid metabolism regulation in chickens, liver tissue was used to detect changes in genes involved in lipid transport. In addition, to investigate the expression patterns of *CEL* during chicken growth, we collected 4 pancreatic tissue samples from different growth stages of AA broilers and LS chickens and measured the expression of the *CEL* gene.

### Cell culture and treatment

The isolation and culture of chicken embryonic primary hepatocytes was performed using the same method as described previously^49,53^. In brief, chicken embryonic primary hepatocytes were seeded at a density of 1 × 10^5^ cells/mL in 6-well plates at 37 °C and 5% CO_2_. Upon reaching 60–80% confluence, the cells were transfected with a *CEL*-overexpression vector (50 nM) or a negative control (50 nM) using 4 μL of Lipofectamine^®^ 2000 (Invitrogen, Carlsbad, CA, USA), and the medium was replaced 6 h later. Cells were collected separately from different treatment groups at 12 h, washed in 1 × PBS (Solarbio, Beijing, China) and collected with TRIzol^®^ reagents (Takara, Kyoto, Japan) and total cellular RNA was extracted to study the regulation of lipid metabolism by the *CEL* gene. All transfections were carried out in triplicate.

### Cloning of the chicken CEL gene and sequencing analysis

The specific P3 primers (Supplementary Table S3) for cloning the coding region (CDS) of the genes were designed using the chicken *CEL* gene and mRNA sequences in GenBank (accession numbers NC_006104 and NM_001012997.1, respectively) with Primer 5.0 software. PCR was performed with a 20 µL final reaction volume containing 10 μL PrimeSTAR^®^ Max DNA Polymerase (Takara, Kyoto, Japan), 1 μL each of forward and reverse primers, and 6 μL RNase-free water. The PCR products were cloned into pMD-18T (Takara, Kyoto, Takara) and sequenced by Sangon Biotech Co. Ltd. (Shanghai, China). The sequence was confirmed by repeating the sequencing three times. Furthermore, to further validate the regulatory role of CEL in cholesterol metabolism *in vitro*, we constructed the recombinant expression plasmid pcDNA3.1-CEL-EGFP. The recombinant vectors were transfected into chicken primary hepatocytes.

### Polymorphism detection and diversity analyses of different breeds

A total of 8 populations were used to identify the 99-bp indel in the 5’ region of the *CEL* gene and for allele frequency analysis. Specific PCR primers P1 (Supplementary Table S3) were designed to amplify the 246/345 bp fragment of the 5’ region of the *CEL* gene to detect the 99-bp insertion variation (GenBank accession NC_006104). Detailed PCR amplification steps refer to previously published literature^54^. Three genotypes were observed, including one genotype with a 345-bp band, one with 246-bp and 345-bp bands and one with a 246-bp band. In addition, the study used the high-throughput sequencing dataset in the NCBI database (http://www.ncbi.nlm.nih.gov/) to explore *CEL* genotype information from two other international breeds of chickens. Indel allele sequences (containing a 99 bp insert) were aligned with clean reads using standard nucleotide BLAST (https://blast.ncbi.nlm.nih.gov/Blast.cgi).

The genotypes, allele frequencies and HWE of the 10 breeds were calculated using the SHEsis program (http://analysis.bio-x.cn). Colony genetic indexes, including heterozygosity (*He*), effective allele numbers (*Ne*) and polymorphism information content (*PIC*), were calculated using Nei’s methods with PopGene software (Version 1.3.1)^55^. The paired fixed index (*Fst*) of feral, native and commercial chickens was tested by GENEPOP 4.5 and Bonferroni correction^56^.

### Total RNA isolation and cDNA preparation

Total RNA was prepared from the different tissues and cells with TRIzol^®^ reagent (Takara, Kyoto, Japan) according to the manufacturer’s protocol, and RNA quality was assessed using electrophoresis and spectrophotometry. Reverse transcription into cDNA was performed using an RNA reverse transcription kit (Takara, Kyoto, Japan) with 2 μg total RNA in a 20 μl reaction.

### Real-time quantitative PCR

Primers spanning exon-exon junctions were synthesized by Sangon Biotech Company (Shanghai, China) for detection of relative expression levels of genes and the *ACTB* (β-actin) gene was used as an internal control. Real-time PCR (RT-PCR) was performed using the SYBR Green method and a Roche LightCycler^®^96 instrument. The composition of the reaction and the amplification step are referred to published in the literature and the reactions were performed in triplicate for each sample. The PCR products were identified via P2-F and P2-R sequencing.

### Bioinformatics analysis

Database searches were performed at NCBI (http://www.ncbi.nlm.nih.gov/) under default settings. The transcriptome data for tissue expression profiling analysis of the *CEL* gene of four species were obtained from the NCBI, and Ensembl databases and previously published articles^57^. The DNA, nucleotide, and amino acid sequences were analyzed using BioXM (Version 2.7) and DNAMAN (version 6.0). Genetic conservation was analyzed using Genomicus available at http://www.genomicus.biologie.ens.fr/genomicus-71.01/cgi-bin/search.pl^49^, which was employed for synteny analysis of genes of interest across multiple species in this study. The secondary structures of the amino acid sequences were predicted at http://smart.embl-heidelberg.de/. The signal peptide and transmembrane helix analyses were performed using online software (http://www.cbs.dtu.dk/ser-vices/SignalP/ and http://www.cbs.dtu.dk/services/TMHMM/, respectively). N-linked glycosylation sites were predicted using NetNGlyc (version 1.0) servers at http://www.cbs.dtu.dk/services/NetN-Glyc/.

### Statistical analysis

Statistical analyses of associations between the genotypes and selected traits of the F_2_ chickens were performed using SPSS 24.0 (SPSS for Windows, Standard version 24.0; SPSS, USA) according to the following two linear mixed models. Model I was used to evaluate growth traits. Considering the effects of body weight on carcass traits, carcass weight was used as a concomitant variable in model II, which was applied to calculate carcass traits^50,54^.

$${\rm{Model}}\,{\rm{I}}:{{\rm{Y}}}_{ijklm}=\mu +{{\rm{G}}}_{i}+{{\rm{S}}}_{j}+{{\rm{H}}}_{k}+{{\rm{f}}}_{l}+{{\rm{e}}}_{ijklm}$$

$${\rm{Model}}\,{\rm{I}}{\rm{I}}:{{\rm{Y}}}_{ijklm}={\rm{\mu }}+{{\rm{G}}}_{i}+{{\rm{S}}}_{j}+{{\rm{H}}}_{k}+{{\rm{f}}}_{l}+{\rm{b}}\,({{\rm{W}}}_{ijklm}-\bar{{\rm{W}}})+{{\rm{e}}}_{ijklm}$$

where Y_*ijklm*_ is the observed value; µ represents the overall population mean; G_*i*_ is the fixed effect of the genotype (i = II, ID, DD); f_*l*_ is the fixed effect of the family (l = 7); S_*j*_ is the fixed effect of sex (j = 2); H_*k*_ is the fixed effect of the hatch (k = 2); b represents the regression coefficient for carcass weight; $\bar{{\rm{W}}}$ is the average slaughter weight; W_*ijklm*_ is individual slaughter weight; and e_*ijklm*_ is the random error. G_*i*_, S_*j*_, H_*k*_ are fixed factors, and f_*l*_ is a random factor. Multiple comparisons were analyzed with the least square means to display significant differences among genotypes with Bonferroni’s test. These trait phenotypic values of different genotypes are expressed as mean ± standard error (SE) and significance was determined by a *p*-value <0.05^58^.

Relative gene expression data and significance in different tissues were analyzed using the 2^−ΔΔCT^ method and one-way analysis of variance (ANOVA) followed by Duncan’s test^59^. The graphics were drawn using GraphPad Prism 7 (GraphPad, San Diego, CA, USA). A *p*-value < 0.05 was considered statistically significant, and a *p*-value < 0.01 was considered highly significant. These data are expressed as the mean ± standard deviation (SD).

## Supplementary information

Supplementary Infomation.

## Data Availability

All data generated or analyzed during this study are included in this published article (and its Supplementary Information Files).
