# Supplementary material for: Association of a new 99-bp indel of the CEL gene promoter region with phenotypic traits in chickens
Source: Sci Rep. 2020 Feb 21;10:3215. doi: 10.1038/s41598-020-60168-2 (PMC7035288; doi:10.1038/s41598-020-60168-2)
Supplement: Supplementary file 1 — Supplementary Infomation. [file 41598_2020_60168_MOESM1_ESM.pdf]

Association of a new 99-bp indel of the *CEL* gene promoter region with phenotypic traits in chickens

Xiangnan Wang#· Xinlei Wang#· Bingjie Chen· Yaping Guo· Hehe Tang· Donghua Li· Danli Liu· Yanbin Wang ·Guoxi Li ·Xiangtao Kang ·Zhuanjian Li*& Ruili Han*

College of Animal Science and Veterinary Medicine, Henan Agricultural University, Zhengzhou, 450046, China

#: Xiangnan Wang and Xinlei Wang contributed equally to this work.

*: Correspondence Author: Zhuanjian Li and Ruili Han

Tel: +86-18037465363 and +86-15188312192

E-mail: lizhuanjian@163.com and rlhan@126.com

Address: College of Animal Science and Veterinary Medicine, Henan Agricultural University, Zhengzhou, 450046, China

Acknowledgments

This study was funded by NSFC-Henan joint grant (U1804107), Program for Innovation Research Team of Ministry of Education (IRT16R23), and the Tackle Key Problem of Science and Technology Project of Henan Province (182102110351).

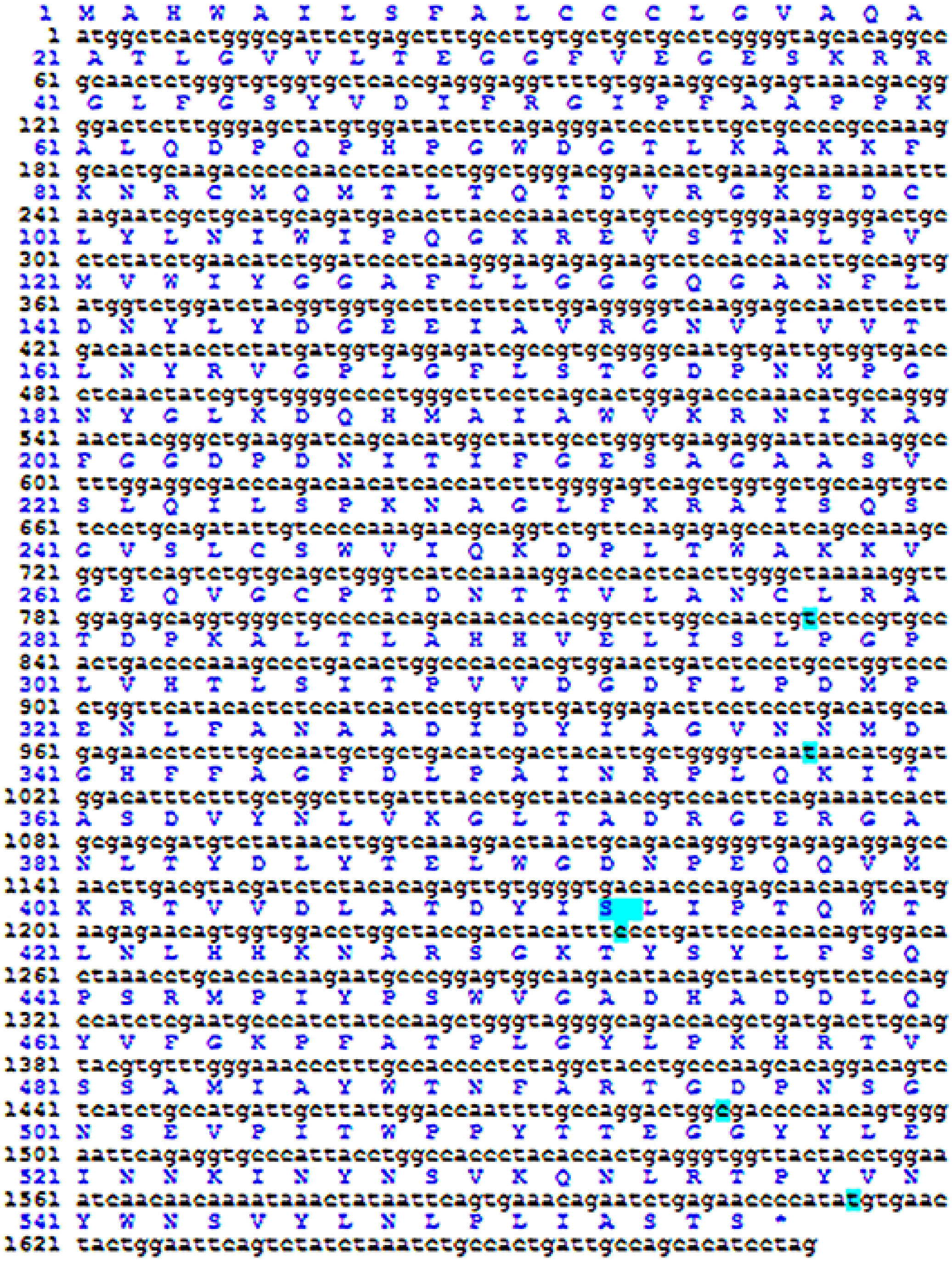

Figure S1. CDS and deduced amino acid sequence for chicken CEL. Nucleotides are shown in lowercase letters, and amino acids are shown in capital letters above the first nucleotide in each codon triplet. Shaded areas are mutation sites.

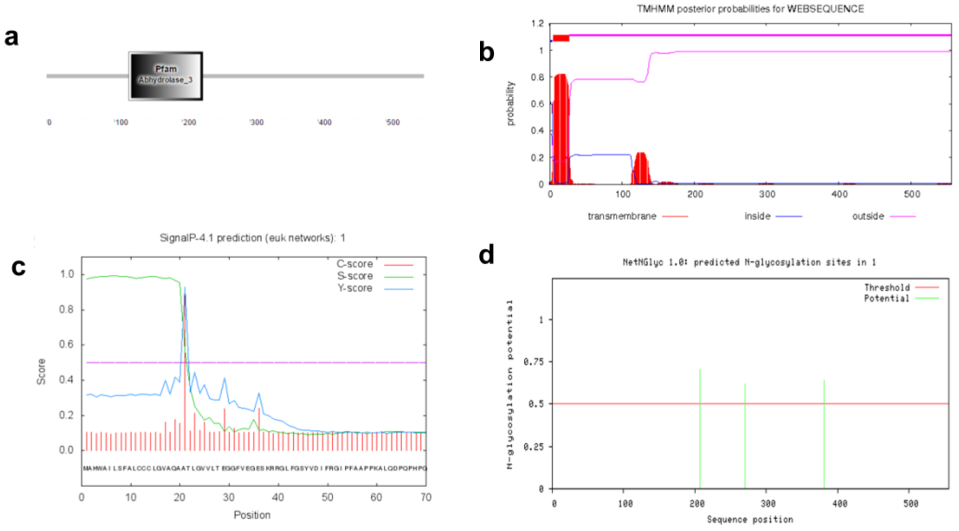

Figure S2. Bioinformatics analyses of CEL amino acid sequences. (a) The secondary structure of the CEL amino acid sequences. (b) TMHMM is the predicted transmembrane region of the CEL amino acid sequence. (c) Results of the CEL signal peptide P4.1 analysis. (d) NetNGlyc 1.0 predicts the glycosylation site of the CEL amino acid sequence.

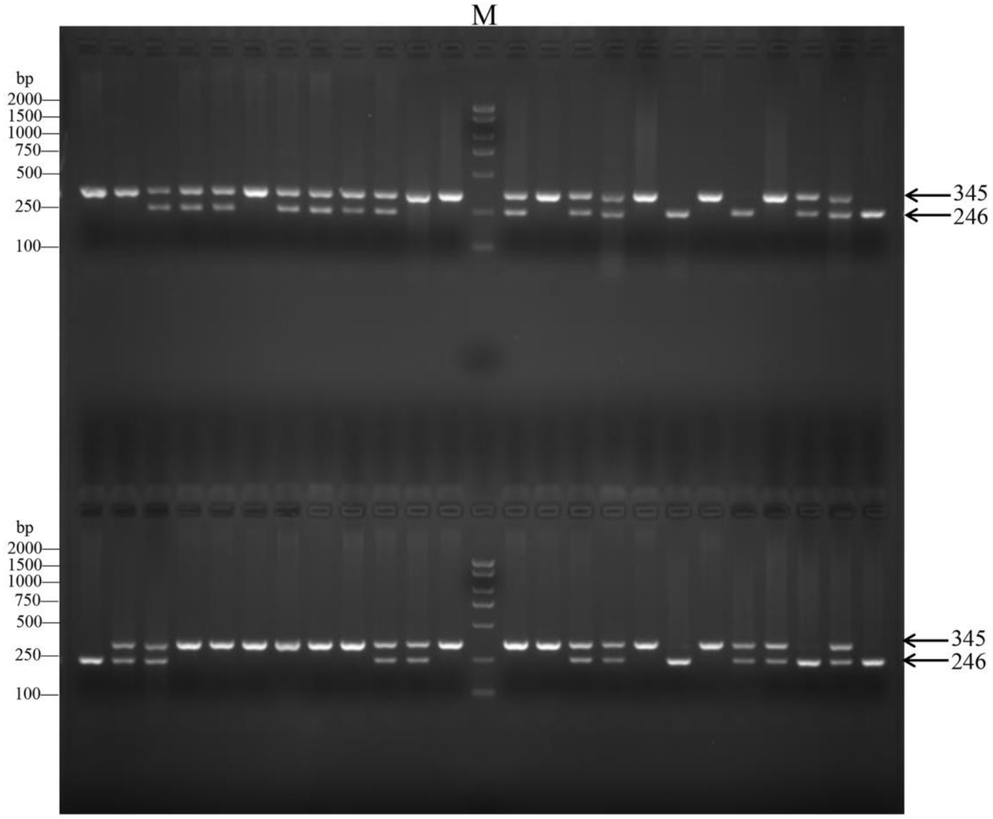

Figure S3. Electrophoresis pattern of the 99-bp indel locus within the chicken *CEL* gene (genotyped by 2.0% agarose gel electrophoresis).

Allele I

GTCACTCAGAGGCTCCCTTTATGGCACGTAACATATATATATGAATAAATATACACACACAGTGTGATCCAGCT
CCTCCTAAAGTATGCATCTAAAATAGGACTGAGAAGTCATGTCCTTAATGCACCTTGTTCTCCCACCGATCAG
AAAGGGAGCCTAGCACATACAGATATCTAAAGTTAcgtgggatgagtcgcactcttgtggtctctgcactgctctcaggctacgtttctcacagct
ggttctgttccagtccacagcctccagctctcctcctgCTCTCCATGCAGAGAGGTTGCTCATGGCCATTTAATTTCTCTGTGTATCG
TGGGTTCCTAGCCA

Allele D

GTCACTCAGAGGCTCCCTTTATGGCACGTAACATATATATATGAATAAATATACACACACAGTGTGATCCAGCT
CCTCCTAAAGTATGCATCTAAAATAGGACTGAGAAGTCATGTCCTTAATGCACCTTGTTCTCCCACCGATCAG
AAAGGGAGCCTAGCACATACAGATATCTAAAGTTACTCTCCATGCAGAGAGGTTGCTCATGGCCATTTAATTT
CTCTGTGTATCGTGGGTTCCTAGCCA

Figure S4. The sequences of alleles I and D. Note: The underlined sequence represents the primer sequence, the lower case base represents the 99-bp insertion sequence, and the blue triangle represents the mutation loci.

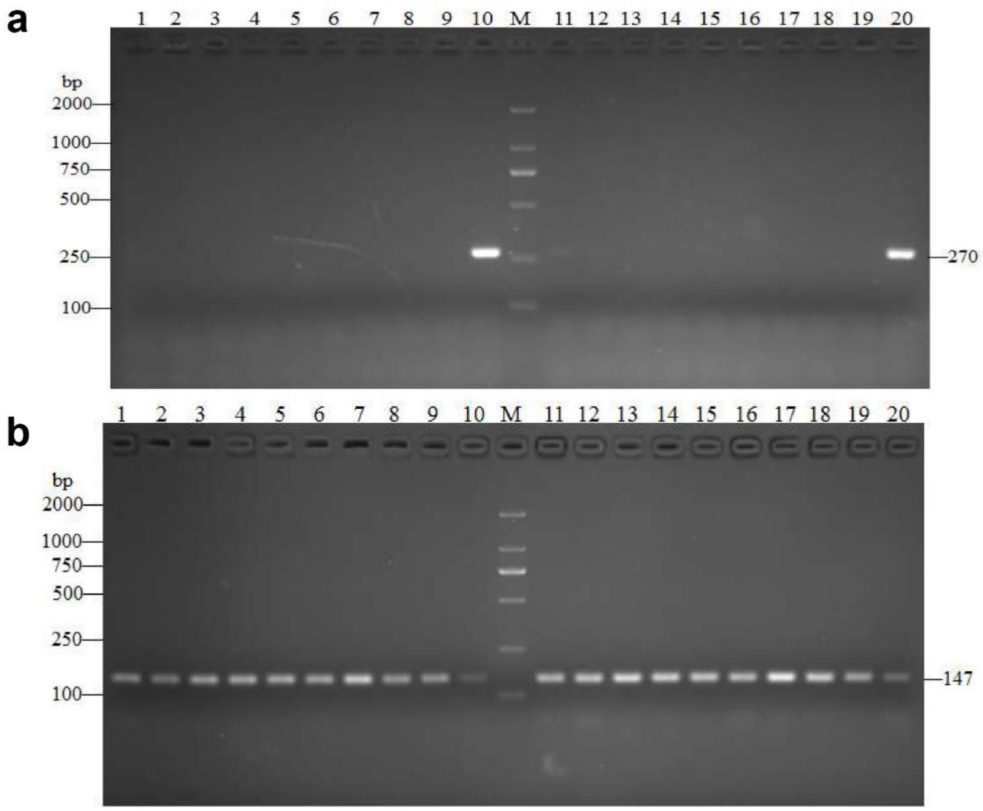

Figure S5. Electrophoresis pattern. (a) Electrophoresis pattern of chicken *CEL* gene expression in 10 tissues of different individuals. (b) Electrophoresis pattern of chicken ACTB gene expression in 10 tissues of different individuals.

Note: Figures 1 to 10 of the **a** and **b** diagrams represent the heart, liver, spleen, lung, kidney, duodenum, ovary, abdominal fat, breast muscle and pancreas of the same individual. Figures 11 to 20 represent ten samples from another individual. Figure. 4a is obtained from the late 11-20 lanes of the **a** and **b** diagrams, respectively.

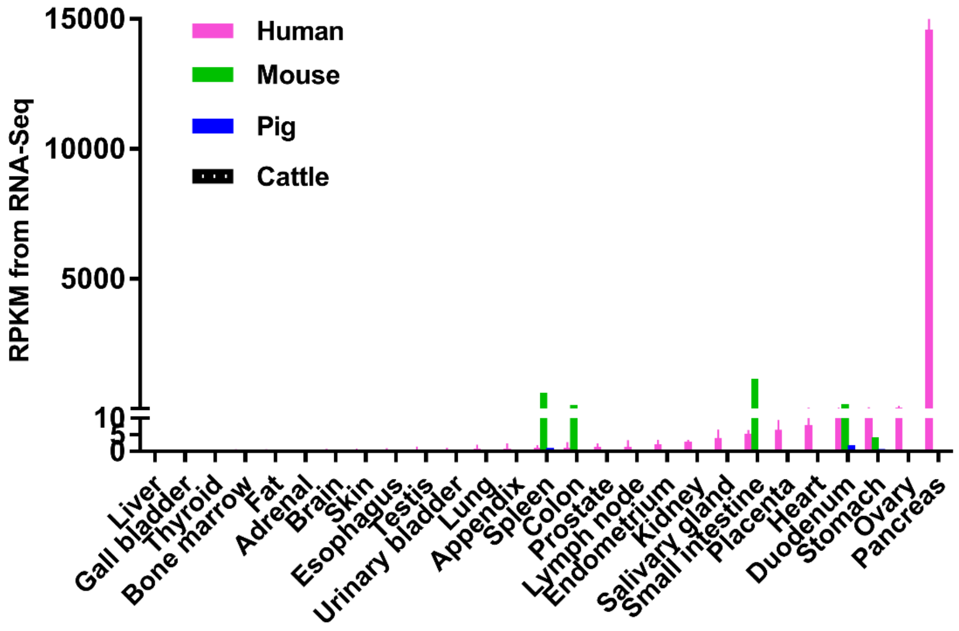

Figure S6. Tissue expression profiles of the *CEL* gene from different species.

Supplementary Table S1. The differences in genotypic frequencies of *χ2* and *P*-values among seven chicken breeds with *CEL* gene mutations

Breeds	XC	LS	CS	DX	Commercial laying hens	Commercial broiler
XC		0.450	4.359	4.031	100.410	172.757
LS	0.798		4.744	2.200	93.660	163.585
CS	0.113	0.093		11.223	118.467	195.664
DX	0.133	0.333	0.004		67.701	123.960
Commercial laying hens	0.000	0.000	0.000	0.000		13.869
Commercial broiler	0.000	0.000	0.000	0.000	0.000	

Note: *χ2* and *P*-value differences for genotypic frequencies between breeds are shown in the uptriangle and the downtriangle of this table, respectively. XC = Xichuan black-bone chicken, LS = Lushi blue-eggshell chicken, CS = Changshun blue-eggshell chicken, DX = Dongxiang blue-eggshell chicken. Commercial laying hens (Hy-Line Brown), commercial broiler (Ross 308 and Arbor Acres broiler).

Supplementary Table S2 Associations of the *CEL* indel with body weight at different stages in chicken

Body weight	（Mean ±SE）			*P*
	II (n=243)	ID (n=415)	DD (n=136)	
BW0(g)	30.46±0.44	30.82±0.42	30.56±0.46	0.210
BW2(g)	122.44±2.57	122.43±2.44	121.59±2.76	0.890
BW4(g)	318.90±6.94	321.85±6.68	327.50±7.42	0.216
BW6(g)	553.95±13.12	562.99±12.60	573.91±13.91	0.092
BW8(g)	806.68±18.79	816.31±18.01	834.50±20.13	0.135
BW10(g)	1099.29±23.63	1110.73±22.65	1139.32±25.16	0.059
BW12(g)	1336.89±27.16	1354.02±25.87	1379.31±29.11	0.121

Note: BW0, 2, 4, 6, 8, 10, and 12 = body weight at the age of 0 days, 2, 4, 6, 8, 10 and 12 weeks, respectively.

Supplementary Table S3. Primers used for amplifying, cloning and expression analysis of chicken the *CEL* gene

Primers	Primer sequences (5′→3′)	Tm	Product size (bp)
P1	F: GTCACTCAGAGGCTCCCTTT	60℃	246/345
	R: TGGCTAGGAACCCACGATAC		
P2	F: CTGGAGACCCAAACATGCCA	60℃	270
	R: CTCTCTTGAACAGACCTGCGT		
P3	F: TGCACACCATGGCTCACTG	55℃	1698
	R: TTTTCAGCGGCTCCACAGTC		
ACTB	F: CAGCCAGCCATGGATGATGA	60℃	147
	R: ACCAACCATCACACCCTGAT		
APOB	F: ATGTTCAAAAGATGCGGCCC	60℃	224
	R: GCATGGCTCTTCTCTCACTG		
MTTP	F: CAGGAGGGATGGAGTTCAGC	60℃	243
	R: TGGTCACGGAATGCCTGAAA		
APOV1	F: CAATGAAACGGCTAGACTCA	60℃	108
	R: AACACCGACTTTTCTTCCAA		
SREBF1	F: GAGACCATCTACAGCTCCGC	60℃	154
	R: CATCCGAAAAGCACCCCTCT		
